# Weibel-Palade bodies – secretory organelles at the interface of inflammation and hemostasis

**DOI:** 10.3389/fcell.2025.1624487

**Published:** 2025-06-19

**Authors:** Julian Terglane, Volker Gerke

**Affiliations:** Institute of Medical Biochemistry, Centre for Molecular Biology of Inflammation, ZMBE, University of Münster, Münster, Germany

**Keywords:** calcium, endothelium, exocytosis, hemostasis, von-Willebrand factor

## Abstract

Weibel-Palade bodies (WPB) are lysosome-related, secretory organelles unique to vascular endothelial cells. They serve as storage organelles for the pro-thrombotic and hemostatic glycoprotein von-Willebrand factor (VWF) as well as numerous other proteins involved in regulating local inflammatory responses and coagulation processes. WPB undergo a complex formation and maturation process mainly dictated by the post-translational maturation of VWF itself. They are born at the trans-Golgi network and then move on microtubules to the cell periphery where they are anchored at the actin cortex to await signals triggering their evoked exocytosis. During this process, VWF undergoes significant compaction that results in an elongated, cigar-like shape of the organelle. WPB also receive material from the endosomal system although the trafficking routes involved here have not been fully unveiled. Exocytosis of WPB is induced by various agonists signaling through intracellular Ca^2+^ or cAMP elevation. It requires mobilization of WPB from the actin cortex and involves a number of docking and fusion mediating protein assemblies. The evoked release of WPB contents converts the endothelial cell surface from a repellant one which permits unrestricted blood flow to an adhesive structure capable of interacting with circulating leukocytes and platelets. Thereby, the endothelium can initiate inflammatory processes and hemostasis when vessel injury has occurred. This review discusses recent developments in the maturation and exocytosis of WPB, focusing on the ionic milieu required for tight VWF packing, endosome-to-WPB transport of WPB cargo, and WPB exocytosis and cargo release.

## Introduction

Vascular endothelial cells not only form a physical barrier separating blood from tissue, they also control vascular homeostasis by secreting factors that regulate coagulation, fibrinolysis and local inflammatory events. These factors include adhesion molecules that enable endothelial cells to rapidly change their surface properties. In resting conditions, the endothelial surface is repulsive and anti-coagulant permitting unrestricted blood flow. However, endothelial activation, e.g., following inflammation/infection or physical blood vessel injury, leads to a rapid transformation and the endothelial surface becomes adhesive towards leukocytes and platelets, supporting among other things platelet aggregation (in injured vessels) and leukocyte extravasation into inflamed tissue. This transition of surface properties is executed by the evoked exocytosis of unique secretory organelles, the Weibel-Palade bodies (WPB), that store the respective platelet and leukocyte adhesion receptors von-Willebrand factor (VWF) and P-selectin, respectively (Weibel and Palade, 1964; Wagner et al., 1982).

WPB are elongated, rod-shaped organelles that are only found in endothelial cells. They undergo a complex maturation process that is dictated by the folding and assembly of its main cargo VWF, a multimeric glycoprotein whose absence or loss of activity is cause of the most common inherited bleeding disorder, von-Willebrand disease (Leebeek and Eikenboom, 2016). Following synthesis in the ER and further processing in the Golgi, multimeric VWF is packaged into immature WPB at the trans-Golgi network (TGN). Further tubulation and tight packing of VWF into paracrystalline assemblies occurs within the organelle; it drives maturation of WPB and establishes the characteristic elongated morphology. Furthermore, maturing WPB acquire several small RabGTPase, most prominently Rab27A, and they receive material from endosomes/lysosomes, such as the P-selectin cofactor CD63. Hence, mature WPB are considered lysosome-related organelles (LRO) (for review see (McCormack et al., 2017; Mourik and Eikenboom, 2017)). The mature, Rab27A-positive WPB are anchored at the cortical actin cytoskeleton and - following endothelial stimulation and a resulting rise in intracellular Ca^2+^ or cAMP concentration - the cortically anchored WPB are transferred to a plasma membrane tethering complex that aids subsequent fusion and cargo release.

In this mini-review we discuss recent developments in WPB biology, published mainly in the last 4 years, focusing on WPB maturation, i.e., the formation of this unique LRO, their exocytosis and specific cargo release.

## The formation of a unique organelle

The characteristic morphology of WPB was already evident in the first EM images of Weibel and Palade (Weibel and Palade, 1964). They appear as elongated rods with an internal pattern of parallel stripes that represent the tightly packed VWF (Zenner et al., 2007; Berriman et al., 2009). VWF is not only the major constituent, it also drives the formation of the unique organelle: endothelial cells lacking VWF fail to produce WPB (Denis et al., 2001; Schillemans et al., 2019) and ectopic expression of VWF induces the formation of WPB-like organelles in non-endothelial cells (Hannah et al., 2003). ProVWF monomers are synthesized in the ER and undergo a sequence of processing steps, which starts with the formation of “tail-to-tail” homodimers via disulfide bridges between the C-terminal CK domains (Voorberg et al., 1991). In the TGN, zipped up proVWF dimers assemble into right-turning helical tubules with the N-terminal domains forming the core and the C-terminal domains spiraling outwards. This tubulation of proVWF is important as it provides the template for multimerization by bringing the D´D3 domains of neighboring dimers in close proximity (Zeng et al., 2022; Anderson et al., 2022; Zhou et al., 2011; Huang et al., 2008; Michaux et al., 2006). Furthermore, the VWF dimers are linked “head-to-head” via formation of N-terminal interdimer disulfide bridges and the propeptide is cleaved, however remains associated with the mature VWF (Zeng et al., 2022; Wagner et al., 1986; Mayadas and Wagner, 1989; Madabhushi et al., 2012; Vischer and Wagner, 1994). Multiple of these multimeric VWF tubules are packed into nascent WPB and upon compaction form paracrystalline arrays in the mature organelle (Zenner et al., 2007; Berriman et al., 2009).

**FIGURE 1:**
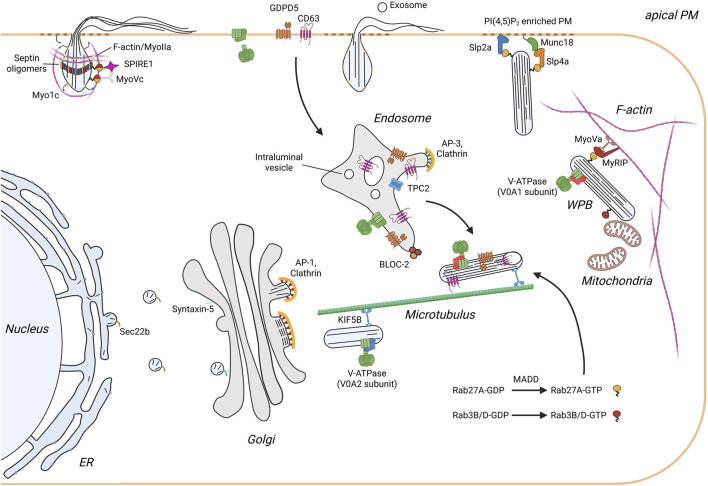
Scheme illustrating the life cycle of WPB in endothelial cells. Monomeric VWF is synthesized into the ER and transported along the anterograde pathway to the Golgi apparatus. This ER/Golgi trafficking requires the SNARE SEC22B, which is present on ER-derived transport vesicles, and the cis Golgi resident SNARE syntaxin 5. In the TGN multiple VWF quanta are incorporated into newly forming WPB. Budding of VWF is initiated by VWF tubules pushing the TGN membrane outward in a process supported by a clathrin coat recruited by the AP-1 complex. The immature WPB are transported along the microtubule network to the cell periphery. During this process WPB undergo maturation which is characterized by V-ATPase mediated acidification, a decrease in width and growth in length, and the appearance of an electron-dense internal structure with incorporated intraluminal vesicles (ILV). Furthermore, additional proteins are recruited. These include proteins of endolysosomal origin such as CD63 that classify WPB as LRO, and cytosolic factors, most importantly Rab27A which links WPB to the actin cytoskeleton and regulates exocytosis through binding of docking/tethering factors. Exocytosis of WPB releases soluble proteins and exosomes into the vascular lumen and presents membrane proteins at the apical surface of the endothelium. Rapid neutralization of WPB upon fusion results in a swelling of the organelle and the spring-loaded unfurling of VWF tubules. Secretion of VWF is additionally supported mechanically by the formation and contraction of an actomyosin ring/coat.

Importantly, VWF maturation and tight packing require a proper ionic milieu, specifically a low pH of appr. 5.5, and, as shown by biochemical studies involving purified VWF domains, high concentrations of Ca^2+^ ions that bind to the protein (Zeng et al., 2022; Anderson et al., 2022; Zhou et al., 2011; Huang et al., 2008; Michaux et al., 2006; Wagner et al., 1986; Mayadas and Wagner, 1989; Madabhushi et al., 2012; Vischer and Wagner, 1994; Erent et al., 2007; Gruber et al., 2022). Recent studies have shed some light on the ionic milieu of WPB and have revealed how this is established. Subunits of the vacuolar proton pump, V-ATPase, have been localized to WPB, first identified in proteomic analyses (van Breevoort et al., 2012; Holthenrich et al., 2019) and later by immunofluorescence approaches and expression of GFP-tagged subunit constructs (Yamazaki et al., 2021; Lu et al., 2021; Terglane et al., 2022). Interestingly, different isoforms of one of the membranous V0 subunits, V0a, were localized to distinct WPB populations (Figure 1). The V0a2 isoform appeared specifically associated with nascent, perinuclear WPB that bud from the TGN, and V0a2 depletion resulted in the formation of WPB with an irregular, twisted morphology. V0a1, on the other hand, was found on mature, peripherally-located and Rab27A-positive organelles. Its absence led to a failure of WPB to bud from the TGN, resulting in lower WPB numbers, which is a bit difficult to reconcile with the peripheral localization of this subunit. At the stage of TGN budding, V0a1 seems to function together with protein kinase D, mediating membrane fission and budding of WPB at the TGN (Yamazaki et al., 2021). A functional involvement of the V-ATPase in establishing/maintaining the low luminal pH of WPB was shown by inhibitor (bafilomycin A) and subunit depletion experiments, which resulted in an altered, less elongated morphology of WPB and defects in secretion of multimeric VWF (Yamazaki et al., 2021; Lu et al., 2021; Terglane et al., 2022).

At present, it is not fully established how a functional V-ATPase is incorporated into the WPB membrane. The enzyme also functions at the Golgi and thereby, could potentially be recruited into nascent WPB budding at the TGN. However, recent evidence indicates that at least some subunits of the enzyme are transported to maturing WPB from endosomal compartments. This conclusion relates to the fact that a subunit of the Biogenesis of Lysosome-related Organelles Complex-2 (BLOC-2) was linked to proper WPB morphology. Specifically, depletion of the Hermansky-Pudlak syndrome (HPS) protein-6 led to misshaped WPB and an impaired tubulation of VWF, which is required for the tight packing of VWF inside WPB and thus, their rod-shaped morphology. A very similar phenotype was observed following knock-down of one of the V-ATPase subunits, V0D1, and the same subunit was shown to interact with HPS6 (Lu et al., 2021). Hence, it appears that V-ATPase subunits are transported via a BLOC-2 dependent pathway from endosomes to the limiting membrane of WPB and that blocking this transport route results in improper V-ATPase assembly and a failure to acidify the lumen of the organelle. A similar endosome-to-WPB trafficking route involving HPS6 was recently also described for the tetraspanin CD63 (Lyososome-Associated Membrane Protein-3, LAMP-3) and the glycerophosphodiester phosphodiesterase domain-containing protein 5 (GDPD5), two other components of the WPB membrane (Sharda et al., 2020; Naß et al., 2024).

The size and morphology of WPB are also affected by other parameters. These include the luminal Ca^2+^ concentration, which was shown to be elevated above cytosolic levels and is most likely required for proper VWF packing in the organelle (Terglane et al., 2025), as well as factors regulating fission/fusion events and WPB transport. Some of these factors recently identified encompass regulators of small GTPases that impact WPB size and consequently, the secretion of multimeric VWF. Following the earlier identification of clathrin and the adapter protein-1 (AP-1) complex as coat proteins supporting the budding of WPB at the TGN (Lui-Roberts et al., 2005), the search for regulators of this process by a targeted siRNA screen yielded two GTPase activating proteins (GAPs) of ADP ribosylation factor (Arf) GTPases. The ArfGAPs were shown to control different steps in the WPB life cycle: SMAP1 was reported to affect WPB size (its depletion results in smaller WPB), most likely though by inhibiting lysosomal degradation of WPB, whereas AGFG2 appeared to promote evoked VWF secretion (Watanabe et al., 2021). However, the mechanisms involved are not fully understood. GAPs typically inactivate their cognate GTPases, while guanine nucleotide exchange factors (GEFs) promote GTP loading and thus activation. GBF1, a known regulator of Arf1 and Arf4, was shown to affect WPB size directly at the level of the Golgi/TGN. Its activity is controlled by phosphorylation via AMP-activated protein kinase 1 (AMPK1) and thereby coupled to the metabolic state. Low glucose treatment of cultured endothelial cells, mimicking the physiological scenario of low blood glucose levels, triggered GBF1 phosphorylation mediated by AMPK1 and in turn a general upregulation of anterograde cargo transport resulting in smaller WPB. Depletion of GBF1, on the other hand, led to oversized WPB that, however, did not acquire the maturation marker Rab27A and were secretion incompetent (Lopes-da-Silva et al., 2019). How Rab27A itself is recruited to the more mature WPB had remained enigmatic for some time but a recent study reported an involvement of the RabGEF, MAP kinase-activating death domain (MADD). MADD was shown to promote nucleotide exchange in Rab27A and the Rab3 isoforms found on WPB, and MADD depletion inhibited recruitment of these Rabs to WPB and consequently, reduced stimulus-evoked VWF secretion (Kat et al., 2021).

Other proteins affecting WPB formation through regulation of membrane trafficking include soluble N-ethylmaleimide sensitive fusion protein attachment protein receptors (SNAREs) found in the ER and Golgi, i.e., organelles of the secretory pathway critically involved in VWF production and maturation. Specifically, the longin-SNARE Sec22b and the v-SNARE syntaxin-5 were shown to control anterograde traffic of VWF and thereby WPB size and morphology. Depletion of Sec22b led to the disintegration of the Golgi, a retention of proVWF in the ER and as a consequence, shorter WPB. Knockdown of syntaxin-5 resulted in similar phenotypes and a reduction in stimulus-evoked VWF secretion (Karampini et al., 2020; Kat et al., 2022). The involvement of ER/Golgi-resident SNAREs in controlling WPB morphology is in line with the important role of Golgi ministacks within the Golgi ribbon that pack VWF multimers into quanta which determine the size of the WPB budding at the TGN (Ferraro et al., 2014; Page et al., 2022). While the involvement of SNAREs underscores the importance of membrane fusion and fission events in ER/Golgi trafficking of VWF and the budding of nascent WPB at the TGN, a role of membrane contact sites in supporting the maturation of WPB is also beginning to emerge. Specifically, frequent contacts between WPB and mitochondria were observed by light and electron microscopy and WPB displayed short and less elongated morphologies when cells were treated with mitochondrial inhibitors. Furthermore, Rab3B was enriched at WPB-mitochondria contacts and depletion of this Rab resulted in a reduction of WPB-mitochondria contacts and shorter WPB indicative of a defect in proper maturation (Ma et al., 2024).

The main WPB cargo, VWF, whose multimerization, tubulation and compaction determines the morphology of WPB, undergoes several modifications within the ER and Golgi. These include N- and O-glycosylations, which play a role in regulating VWF activities in the vasculature and also post-translational processing of the VWF itself (Ward et al., 2021; Preston et al., 2013; O’Sullivan et al., 2016). While N-linked glycosylation had been shown to affect multimerization of VWF (McKinnon et al., 2010), inhibition of O-linked glycolysation was recently reported to result in shorter and rounder WPB, together with a reduction in VWF multimerization and stimulus-evoked secretion. Interestingly, WPB targeting of another O-glycosylated cargo, the Tie-2 tyrosine kinase ligand angiopoietin-2, was not affected in inhibitor-treated cells although its secretion was altered like that of VWF (Karampini et al., 2024). Similar to several other constituents of WPB, angiopoietin-2 (Ang-2) is most likely targeted to the organelle via interaction with VWF and experiments with isolated VWF derivatives suggest that the D’D3, A1 and A2 domains serve as major binding sites. Furthermore, it was shown that high Ca^2+^ concentrations and a slightly acidic pH promote the interaction in line with the importance of this ionic milieu in supporting WPB maturation (Mobayen et al., 2023; Texier et al., 2023).

The picture emerging from these recent studies underscores the complexity of WPB formation and maturation. It is driven by the processing of VWF itself which requires a low pH and high Ca^2+^ levels in the organelle. Establishment/maintenance of the low pH is dependent on a crosstalk with the endosomal system through trafficking of V-ATPase subunits. Other cargo is also delivered via this route, most notably the P-selectin cofactor CD63, whose transport requires activity of the endosomal two pore Ca^2+^ channel, TPC2 (Goretzko et al., 2023), and the phosphodiesterase GDPD5, which upon WPB exocytosis removes a specific subset of glycosylphosphatidylinositol (GPI)-anchored proteins from the surface of activated endothelial cells (Naß et al., 2024). Together, the VWF maturation/packaging steps and the delivery of cargo from the endosomal system establish the mature WPB.

## Evoked exocytosis of a very large lysosome related organelle

Following formation of nascent WPB and their maturation (see above), the organelles are anchored in the cell periphery awaiting a secretory stimulus. The stimuli include inflammatory activators like thrombin and histamine, which through their respective receptors initiate intracellular Ca^2+^ elevation (Hamilton and Sims, 1987). Secretagogues such as vasopressin also induce WPB exocytosis and they act via cAMP dependent signaling, but this pathway has been studied less extensively than the Ca^2+^ evoked exocytosis (Vischer and Wollheim, 1997). To exocytose, WPB first have to be mobilized from anchorage at the cortical cytoskeleton. While the regulation and molecular mechanism of this release step remain to be fully described, the subsequent steps in the exocytotic response, i.e., docking/tethering of WPB at the plasma membrane, their fusion and the release of cargo, have been assessed in more detail. Here, we will summarize recent advance towards understanding the complexity and unique features of these steps.

Mature WPB are highly elongated, rod-like structures and it has been shown recently that they preferentially initiate fusion with the plasma membrane at their tips, a topology also seen in earlier cryo-EM studies (Berriman et al., 2009). Such tip-end fusion involving a more strongly curved part of the organelle membrane is probably supportive of the actual membrane fusion process but also permits a more selective release of the ultra-large hemostatic VWF as opposed to smaller soluble cargo (see below). A screen for mediators of this topological orientation during the tethering and later fusion step identified a Rab27A effector, the C2 domain containing protein synaptotagmin-like protein 2-a (Slp2a), which was shown to act as a positive regulator of WPB exocytosis. Upon secretagogue stimulation with Ca^2+^ mobilizing agonists, Slp2a became enriched at the WPB tip that later underwent fusion and this selective enrichment was dependent on the capacity of Slp2a to bind the membrane lipid phosphatidylinositol 4,5 bisphosphate [PI(4,5)P_2_] (Naß et al., 2022). PI(4,5)P_2_ was earlier shown to accumulate at WPB fusion sites and to be required for efficient VWF secretion (Nguyen et al., 2020). The model emerging from these studies suggests that Slp2a associates with mature WPB via binding of its Slp homology domain to Rab27A and also its C2AB domain to phosphatidylserine (PS) present in the WPB membrane. Following secretagogue-evoked Ca^2+^ increase, the PS binding is weakened allowing the C2AB domain to interact with plasma membrane PI(4,5)P_2_ thus yielding an enrichment of Slp2a at the tip of the organelle that is closest to the plasma membrane (Naß et al., 2022). Slp2a has also been shown to promote WPB exocytosis in more complex tissue culture and animal models of vascular lumen formation. Through interaction with PI(4,5)P_2_, Slp2a associated with the apical membrane during lumen initiation and expansion, and deletion of the PI(4,5)P_2_ binding C2AB domain resulted in an exclusive WPB localization. Furthermore, Slp2a depletion inhibited WPB exocytosis and severely compromised lumen formation, a phenotype that could be rescued with wild-type Slp2a but not with mutants lacking C2AB. The apically polarized, Slp2a-dependent exocytosis of WPB released Ang-2 as WPB cargo which in turn was shown to mediate vascular lumen formation via activation of Tie-2 signaling (Francis et al., 2021).

Other tethering/docking factors most likely cooperate with Slp2a in the course of WPB exocytosis. They include Munc13-4, which upon secretagogue stimulation docks to the annexin A2-S100A10 complex concentrated at PI(4,5)P_2_- and phosphatidic acid-rich plasma membrane domains (Chehab et al., 2017), and the Rab27A effector Slp4, which competes with MyRIP for occupancy at WPB-associated Rab27A and thereby possibly supports the release of cortically anchored WPB in secretagogue stimulated cells (Bierings et al., 2012). While several other Rab proteins have been reported earlier to promote WPB exocytosis, in some cases cooperating with Arf family members (Zografou et al., 2012; Biesemann et al., 2017), small GTPases of the Ral family have also been linked to the final steps of regulated WPB exocytosis. Ral and its nucleotide exchange factor RalGDS were shown earlier to positively stimulate VWF secretion from WPB (Rondaij et al., 2008; de Leeuw et al., 2001). A recent study now addressed the mechanism of Ral action revealing that RalB but not RalA supports WPB exocytosis. In its GDP-bound conformation, RalB interacted with the exocyst complex in resting endothelial cells, and endothelial activation resulted in nucleotide exchange and the uncoupling of RalB-GTP from exocyst. This in turn triggered WPB tethering at the plasma membrane and membrane fusion suggesting an important role of RalB in regulating the WPB tethering step, possibly in conjunction with the above-mentioned tethering factors (Yang et al., 2025).

Following fusion with the plasma membrane, large VWF multimers require, at least in some scenarios, additional forces to be released from WPB into the vasculature. Actomyosin-containing rings/coats have been reported to form around fused WPB that contract to squeeze out ultra-large VWF multimers whereas this contraction appears to be dispensable for the release of smaller cargo such as P-selectin (Nightingale et al., 2018; Miklavc and Frick, 2020). A recent proximity proteomics approach, designed to identify factors specifically associated with Rab27A-positive WPB following secretagogue stimulation, yielded several actin-binding proteins that could support this process (El-Mansi et al., 2023). These included the class I myosin Myo1c which was shown to be recruited to fused WPB via its PI(4,5)P_2_ binding pleckstrin homology domain (El-Mansi et al., 2024), in line with the enrichment of this lipid at WPB fusion sites (see above). Interestingly, Myo1c enrichment occurred in an actin-independent manner and inhibition and depletion experiments combined with live cell imaging indicated that Myo1c most likely supports VWF expulsion from fused WPB by providing additional traction points for the actin ring/coat (El-Mansi et al., 2024).

Conventional myosin motors have also been implicated in actin ring formation/stabilization and VWF expulsion, specifically non-muscle myosin IIA and IIB and myosin Vc (Holthenrich et al., 2022; Nightingale et al., 2011; Li et al., 2018). However, how the polymeric actin rings assemble at the WPB membrane following fusion is not fully understood. Lipids enriched at the fusion site such as PI(4,5)P_2_ could be involved in recruiting actin binding proteins including those that could initiate actin polymerization exactly at this site. Alternatively, actin nucleation promoting factors could be present on WPB before exocytosis and could be specifically activated once secretagogue-evoked fusion occurred. The latter likely holds true for Spire1 which is recruited to mature WPB via Rab27A binding and following histamine-induced WPB fusion with the plasma membrane, supports actin ring formation and the release of large VWF multimers (Holthenrich et al., 2022). Another level of regulation of actomyosin ring assembly was also identified recently. Members of the septin family of GTPases involved in cytoskeleton regulation were shown to localize as ring-like structures to fused WPB. The septin rings form independently of actin polymerization and septin inhibition did not perturb actin recruitment to fused WPB. Recruitment of septin oligomers into the ring-like structures was shown to require the activity of p21-activated kinase 2 (PAK2) suggesting a role of the kinase in the upstream regulation. Septin inhibition reduced the release of large VWF multimers and prolonged the lifetime of actin rings. Thus, two ring-like structures appear to form at WPB post fusion with the septin ring regulating the dynamics and efficiency of actomyosin contraction that expels ultra-large VWF (El-Mansi et al., 2023).

## Concluding remarks

WPB are lysosome-related organelles unique to vascular endothelial cells. They respond rapidly to secretagogue stimulation by Ca^2+^- or cAMP-evoked exocytosis resulting in the release of factors that control hemostasis and inflammation. Work in recent years has revealed exciting novel aspects of WPB biogenesis and secretion that include transport routes from endosomes to maturing WPB and mechanisms underlying the assembly and regulation of an actomyosin ring that forms at WPB post fusion with the plasma membrane and supports the release of ultra-large VWF. However, important questions concerning WPB biology still remain to be answered. They include the regulation of actomyosin assembly and activity and also molecular aspects of the endosome-to-WPB transport route, e.g., do different such transport pathways exist for different WPB cargo such as V-ATPase subunits, CD63, and GDPD5, and does transfer occur via vesicular intermediates or transient organelle fusion. Advances along these lines could benefit the development of pharmacological strategies tailored to controlling the release of inflammatory (e.g., P-selectin) vs. thrombotic WPB cargo (VWF) in pathophysiological situations.

## References

[B1] Anderson J. R. Li J. Springer T. A. Brown A. (2022). Structures of VWF tubules before and after concatemerization reveal a mechanism of disulfide bond exchange. Blood 140 (12), 1419–1430. 10.1182/blood.2022016467 35776905 PMC9507011

[B2] Berriman J. A. Li S. Hewlett L. J. Wasilewski S. Kiskin F. N. Carter T. (2009). Structural organization of Weibel-Palade bodies revealed by cryo-EM of vitrified endothelial cells. Proc. Natl. Acad. Sci. U. S. A. 106 (41), 17407–17412. 10.1073/pnas.0902977106 19805028 PMC2765093

[B3] Bierings R. Hellen N. Kiskin N. Knipe L. Fonseca A. V. Patel B. (2012). The interplay between the Rab27A effectors Slp4-a and MyRIP controls hormone-evoked Weibel-Palade body exocytosis. Blood 120 (13), 2757–2767. 10.1182/blood-2012-05-429936 22898601 PMC3501642

[B4] Biesemann A. Gorontzi A. Barr F. Gerke V. (2017). Rab35 protein regulates evoked exocytosis of endothelial Weibel-Palade bodies. J. Biol. Chem. 292 (28), 11631–11640. 10.1074/jbc.M116.773333 28566286 PMC5512060

[B5] Chehab T. Santos N. C. Holthenrich A. Koerdt S. N. Disse J. Schuberth C. (2017). A novel Munc13-4/S100A10/annexin A2 complex promotes Weibel-Palade body exocytosis in endothelial cells. Mol. Biol. Cell 28 (12), 1688–1700. 10.1091/mbc.E17-02-0128 28450451 PMC5469611

[B6] de Leeuw H. P. Fernandez-Borja M. Reits E. A. Romani de Wit T. Wijers-Koster P. M. Hordijk P. L. (2001). Small GTP-binding protein Ral modulates regulated exocytosis of von Willebrand factor by endothelial cells. Arterioscler. Thromb. Vasc. Biol. 21 (6), 899–904. 10.1161/01.atv.21.6.899 11397694

[B7] Denis C. V. André P. Saffaripour S. Wagner D. D. (2001). Defect in regulated secretion of P-selectin affects leukocyte recruitment in von Willebrand factor-deficient mice. Proc. Natl. Acad. Sci. U. S. A. 98 (7), 4072–4077. 10.1073/pnas.061307098 11274431 PMC31181

[B8] El-Mansi S. Mitchell T. P. Mobayen G. McKinnon T. A. J. Miklavc P. Frick M. (2024). Myosin-1C augments endothelial secretion of von Willebrand factor by linking contractile actomyosin machinery to the plasma membrane. Blood Adv. 8 (17), 4714–4726. 10.1182/bloodadvances.2024012590 38669344 PMC11413703

[B9] El-Mansi S. Robinson C. L. Kostelnik K. B. McCormack J. J. Mitchell T. P. Lobato-Márquez D. (2023). Proximity proteomics identifies septins and PAK2 as decisive regulators of actomyosin-mediated expulsion of von Willebrand factor. Blood 141 (8), 930–944. 10.1182/blood.2022017419 36564030 PMC10023740

[B10] Erent M. Meli A. Moisoi N. Babich V. Hannah M. J. Skehel P. (2007). Rate, extent and concentration dependence of histamine-evoked Weibel-Palade body exocytosis determined from individual fusion events in human endothelial cells. J. Physiol. 583 (Pt 1), 195–212. 10.1113/jphysiol.2007.132993 17540703 PMC2277235

[B11] Ferraro F. Kriston-Vizi J. Metcalf D. J. Martin-Martin B. Freeman J. Burden J. J. (2014). A two-tier Golgi-based control of organelle size underpins the functional plasticity of endothelial cells. Dev. Cell 29 (3), 292–304. 10.1016/j.devcel.2014.03.021 24794632 PMC4022834

[B12] Francis C. R. Claflin S. Kushner E. J. (2021). Synaptotagmin-like protein 2a regulates angiogenic lumen formation via Weibel-Palade body apical secretion of angiopoietin-2. Arterioscler. Thromb. Vasc. Biol. 41 (6), 1972–1986. 10.1161/ATVBAHA.121.316113 33853352 PMC8159857

[B13] Goretzko J. Pauels I. Heitzig N. Thomas K. Kardell M. Naß J. (2023). P-selectin-dependent leukocyte adhesion is governed by endolysosomal two-pore channel 2. Cell Rep. 42 (12), 113501. 10.1016/j.celrep.2023.113501 38039128

[B14] Gruber S. Löf A. Hausch A. Kutzki F. Jöhr R. Obser T. (2022). A conformational transition of the D′D3 domain primes von Willebrand factor for multimerization. Blood Adv. 6 (17), 5198–5209. 10.1182/bloodadvances.2022006978 36069828 PMC9631632

[B15] Hamilton K. K. Sims P. J. (1987). Changes in cytosolic Ca2+ associated with von Willebrand factor release in human endothelial cells exposed to histamine. Study of microcarrier cell monolayers using the fluorescent probe indo-1. J. Clin. Invest 79 (2), 600–608. 10.1172/JCI112853 3492515 PMC424139

[B16] Hannah M. J. Hume A. N. Arribas M. Williams R. Hewlett L. J. Seabra M. C. (2003). Weibel-Palade bodies recruit Rab27 by a content-driven, maturation-dependent mechanism that is independent of cell type. J. Cell Sci. 116 (Pt 19), 3939–3948. 10.1242/jcs.00711 12928333

[B17] Holthenrich A. Drexler H. C. A. Chehab T. Naß J. Gerke V. (2019). Proximity proteomics of endothelial Weibel-Palade bodies identifies novel regulator of von Willebrand factor secretion. Blood 134 (12), 979–982. 10.1182/blood.2019000786 31262780 PMC8270391

[B18] Holthenrich A. Terglane J. Naß J. Mietkowska M. Kerkhoff E. Gerke V. (2022). Spire1 and Myosin Vc promote Ca2+-evoked externalization of von Willebrand factor in endothelial cells. Cell Mol. Life Sci. CMLS 79 (2), 96. 10.1007/s00018-021-04108-x 35084586 PMC8794916

[B19] Huang R. H. Wang Y. Roth R. Yu X. Purvis A. R. Heuser J. E. (2008). Assembly of Weibel-Palade body-like tubules from N-terminal domains of von Willebrand factor. Proc. Natl. Acad. Sci. U. S. A. 105 (2), 482–487. 10.1073/pnas.0710079105 18182488 PMC2206562

[B20] Karampini E. Bürgisser P. E. Olins J. Mulder A. A. Jost C. R. Geerts D. (2020). Sec22b determines Weibel-Palade body length by controlling anterograde ER-Golgi transport. Haematologica 106 (4), 1138–1147. 10.3324/haematol.2019.242727 PMC8018124 32336681

[B21] Karampini E. Doherty D. Bürgisser P. E. Garre M. Schoen I. Elliott S. (2024). O-glycan determinants regulate VWF trafficking to Weibel-Palade bodies. Blood Adv. 8 (12), 3254–3266. 10.1182/bloodadvances.2023012499 38640438 PMC11226974

[B22] Kat M. Bürgisser P. E. Janssen H. De Cuyper I. M. Conte I. L. Hume A. N. (2021). GDP/GTP exchange factor MADD drives activation and recruitment of secretory Rab GTPases to Weibel-Palade bodies. Blood Adv. 5 (23), 5116–5127. 10.1182/bloodadvances.2021004827 34551092 PMC9153003

[B23] Kat M. Karampini E. Hoogendijk A. J. Bürgisser P. E. Mulder A. A. Van Alphen F. P. J. (2022). Syntaxin 5 determines Weibel-Palade body size and von Willebrand factor secretion by controlling Golgi architecture. Haematologica 107 (8), 1827–1839. 10.3324/haematol.2021.280121 35081689 PMC9335113

[B24] Leebeek F. W. G. Eikenboom J. C. J. (2016). Von willebrand’s disease. N. Engl. J. Med. 375 (21), 2067–2080. 10.1056/NEJMra1601561 27959741

[B25] Li P. Wei G. Cao Y. Deng Q. Han X. Huang X. (2018). Myosin IIa is critical for cAMP-mediated endothelial secretion of von Willebrand factor. Blood 131 (6), 686–698. 10.1182/blood-2017-08-802140 29208598

[B26] Lopes-da-Silva M. McCormack J. J. Burden J. J. Harrison-Lavoie K. J. Ferraro F. Cutler D. F. (2019). A GBF1-dependent mechanism for environmentally responsive regulation of ER-golgi transport. Dev. Cell 49 (5), 786–801.e6. 10.1016/j.devcel.2019.04.006 31056345 PMC6764485

[B27] Lu J. Ma J. Hao Z. Li W. (2021). HPS6 regulates the biogenesis of weibel-palade body in endothelial cells through trafficking v-ATPase to its limiting membrane. Front. Cell Dev. Biol. 9, 743124. 10.3389/fcell.2021.743124 35252216 PMC8891752

[B28] Lui-Roberts W. W. Y. Collinson L. M. Hewlett L. J. Michaux G. Cutler D. F. (2005). An AP-1/clathrin coat plays a novel and essential role in forming the Weibel-Palade bodies of endothelial cells. J. Cell Biol. 170 (4), 627–636. 10.1083/jcb.200503054 16087708 PMC2171491

[B29] Ma J. Hao Z. Zhang Y. Li L. Huang X. Wang Y. (2024). Physical contacts between mitochondria and WPBs participate in WPB maturation. Arterioscler. Thromb. Vasc. Biol. 44 (1), 108–123. 10.1161/ATVBAHA.123.319939 37942609

[B30] Madabhushi S. R. Shang C. Dayananda K. M. Rittenhouse-Olson K. Murphy M. Ryan T. E. (2012). von Willebrand factor (VWF) propeptide binding to VWF D’D3 domain attenuates platelet activation and adhesion. Blood 119 (20), 4769–4778. 10.1182/blood-2011-10-387548 22452980 PMC3367877

[B31] Mayadas T. N. Wagner D. D. (1989). *In vitro* Multimerization of von Willebrand Factor Is Triggered by Low pH. J. Biol. Chem. 264 (23), 13497–13503. 10.1016/s0021-9258(18)80024-2 2788160

[B32] McCormack J. J. Lopes da Silva M. Ferraro F. Patella F. Cutler D. F. (2017). Weibel-Palade bodies at a glance. J. Cell Sci. 130 (21), 3611–3617. 10.1242/jcs.208033 29093059

[B33] McKinnon T. A. J. Goode E. C. Birdsey G. M. Nowak A. A. Chan A. C. K. Lane D. A. (2010). Specific N-linked glycosylation sites modulate synthesis and secretion of von Willebrand factor. Blood 116 (4), 640–648. 10.1182/blood-2010-02-267450 20418283

[B34] Michaux G. Abbitt K. B. Collinson L. M. Haberichter S. L. Norman K. E. Cutler D. F. (2006). The physiological function of von Willebrand’s factor depends on its tubular storage in endothelial Weibel-Palade bodies. Dev. Cell 10 (2), 223–232. 10.1016/j.devcel.2005.12.012 16459301

[B35] Miklavc P. Frick M. (2020). Actin and myosin in non-neuronal exocytosis. Cells 9 (6), 1455. 10.3390/cells9061455 32545391 PMC7348895

[B36] Mobayen G. Smith K. Ediriwickrema K. Starke R. D. Solomonidis E. G. Laffan M. A. (2023). von Willebrand factor binds to angiopoietin-2 within endothelial cells and after release from Weibel-Palade bodies. J. Thromb. Haemost. JTH 21 (7), 1802–1812. 10.1016/j.jtha.2023.03.027 37011710

[B37] Mourik M. Eikenboom J. (2017). Lifecycle of weibel-palade bodies. Hamostaseologie 37 (1), 13–24. 10.5482/HAMO-16-07-0021 28004844

[B38] Naß J. Koerdt S. N. Biesemann A. Chehab T. Yasuda T. Fukuda M. (2022). Tip-end fusion of a rod-shaped secretory organelle. Cell Mol. Life Sci. CMLS 79 (6), 344. 10.1007/s00018-022-04367-2 35660980 PMC9167223

[B39] Naß J. Terglane J. Zeuschner D. Gerke V. (2024). Evoked weibel-palade body exocytosis modifies the endothelial cell surface by releasing a substrate-selective phosphodiesterase. Adv. Sci. Weinh Baden-Wurtt Ger. 11 (16), e2306624. 10.1002/advs.202306624 PMC11040351 38359017

[B40] Nguyen T. T. N. Koerdt S. N. Gerke V. (2020). Plasma membrane phosphatidylinositol (4,5)-bisphosphate promotes Weibel-Palade body exocytosis. Life Sci. Alliance 3 (11), e202000788. 10.26508/lsa.202000788 32826291 PMC7442956

[B41] Nightingale T. D. McCormack J. J. Grimes W. Robinson C. Lopes da Silva M. White I. J. (2018). Tuning the endothelial response: differential release of exocytic cargos from Weibel-Palade bodies. J. Thromb. Haemost. 16 (9), 1873–1886. 10.1111/jth.14218 29956444 PMC6166140

[B42] Nightingale T. D. White I. J. Doyle E. L. Turmaine M. Harrison-Lavoie K. J. Webb K. F. (2011). Actomyosin II contractility expels von Willebrand factor from Weibel-Palade bodies during exocytosis. J. Cell Biol. 194 (4), 613–629. 10.1083/jcb.201011119 21844207 PMC3160584

[B43] O’Sullivan J. M. Aguila S. McRae E. Ward S. E. Rawley O. Fallon P. G. (2016). N-linked glycan truncation causes enhanced clearance of plasma-derived von Willebrand factor. J. Thromb. Haemost. JTH 14 (12), 2446–2457. 10.1111/jth.13537 27732771

[B44] Page K. M. McCormack J. J. Lopes-da-Silva M. Patella F. Harrison-Lavoie K. Burden J. J. (2022). Structure modeling hints at a granular organization of the Golgi ribbon. BMC Biol. 20 (1), 111. 10.1186/s12915-022-01305-3 35549945 PMC9102599

[B45] Preston R. J. S. Rawley O. Gleeson E. M. O’Donnell J. S. (2013). Elucidating the role of carbohydrate determinants in regulating hemostasis: insights and opportunities. Blood 121 (19), 3801–3810. 10.1182/blood-2012-10-415000 23426946

[B46] Rondaij M. G. Bierings R. van Agtmaal E. L. Gijzen K. A. Sellink E. Kragt A. (2008). Guanine exchange factor RalGDS mediates exocytosis of Weibel-Palade bodies from endothelial cells. Blood 112 (1), 56–63. 10.1182/blood-2007-07-099309 18417737

[B47] Schillemans M. Kat M. Westeneng J. Gangaev A. Hofman M. Nota B. (2019). Alternative trafficking of Weibel-Palade body proteins in CRISPR/Cas9-engineered von Willebrand factor-deficient blood outgrowth endothelial cells. Res. Pract. Thromb. Haemost. 3 (4), 718–732. 10.1002/rth2.12242 31624792 PMC6782018

[B48] Sharda A. V. Barr A. M. Harrison J. A. Wilkie A. R. Fang C. Mendez L. M. (2020). VWF maturation and release are controlled by 2 regulators of Weibel-Palade body biogenesis: exocyst and BLOC-2. Blood 136 (24), 2824–2837. 10.1182/blood.2020005300 32614949 PMC7731791

[B49] Terglane J. Menche D. Gerke V. (2022). Acidification of endothelial Weibel-Palade bodies is mediated by the vacuolar-type H+-ATPase. PloS One 17 (6), e0270299. 10.1371/journal.pone.0270299 35767558 PMC9242466

[B50] Terglane J. Mertes N. Weischer S. Zobel T. Johnsson K. Gerke V. (2025). Chemigenetic Ca2+ indicators report elevated Ca2+ levels in endothelial Weibel-Palade bodies. PLOS ONE 20 (1), e0316854. 10.1371/journal.pone.0316854 39869616 PMC11771901

[B51] Texier A. Lenting P. J. Denis C. V. Roullet S. Christophe O. D. (2023). Angiopoietin-2 binds to multiple interactive sites within von Willebrand factor. Res. Pract. Thromb. Haemost. 7 (7), 102204. 10.1016/j.rpth.2023.102204 37854453 PMC10579536

[B52] van Breevoort D. van Agtmaal E. L. Dragt B. S. Gebbinck J. K. Dienava-Verdoold I. Kragt A. (2012). Proteomic screen identifies IGFBP7 as a novel component of endothelial cell-specific Weibel-Palade bodies. J. Proteome Res. 11 (5), 2925–2936. 10.1021/pr300010r 22468712

[B53] Vischer U. M. Wagner D. D. (1994). von Willebrand factor proteolytic processing and multimerization precede the formation of Weibel-Palade bodies. Blood 83 (12), 3536–3544. 10.1182/blood.v83.12.3536.3536 8204880

[B54] Vischer U. M. Wollheim C. B. (1997). Epinephrine induces von Willebrand factor release from cultured endothelial cells: involvement of cyclic AMP-dependent signalling in exocytosis. Thromb. Haemost. 77 (6), 1182–1188. 10.1055/s-0038-1656135 9241755

[B55] Voorberg J. Fontijn R. Calafat J. Janssen H. van Mourik J. A. Pannekoek H. (1991). Assembly and routing of von Willebrand factor variants: the requirements for disulfide-linked dimerization reside within the carboxy-terminal 151 amino acids. J. Cell Biol. 113 (1), 195–205. 10.1083/jcb.113.1.195 2007623 PMC2288914

[B56] Wagner D. D. Mayadas T. Marder V. J. (1986). Initial glycosylation and acidic pH in the Golgi apparatus are required for multimerization of von Willebrand factor. J. Cell Biol. 102 (4), 1320–1324. 10.1083/jcb.102.4.1320 3082891 PMC2114173

[B57] Wagner D. D. Olmsted J. B. Marder V. J. (1982). Immunolocalization of von Willebrand protein in Weibel-Palade bodies of human endothelial cells. J. Cell Biol. 95 (1), 355–360. 10.1083/jcb.95.1.355 6754744 PMC2112360

[B58] Ward S. O’Sullivan J. M. O’Donnell J. S. (2021). The Biological Significance of von Willebrand Factor O-Linked Glycosylation. Semin. Thromb. Hemost. 47 (7), 855–861. 10.1055/s-0041-1726373 34130346

[B59] Watanabe A. Hataida H. Inoue N. Kamon K. Baba K. Sasaki K. (2021). Arf GTPase-activating proteins SMAP1 and AGFG2 regulate the size of Weibel-Palade bodies and exocytosis of von Willebrand factor. Biol. Open 10 (9), bio058789. 10.1242/bio.058789 34369554 PMC8430232

[B60] Weibel E. R. Palade G. E. (1964). New cytoplasmic components in arterial endothelia. J. Cell Biol. 23 (1), 101–112. 10.1083/jcb.23.1.101 14228505 PMC2106503

[B61] Yamazaki Y. Eura Y. Kokame K. (2021). V-ATPase V0a1 promotes Weibel-Palade body biogenesis through the regulation of membrane fission. eLife 10, e71526. 10.7554/eLife.71526 34904569 PMC8718113

[B62] Yang M. Boye-Doe A. Ali M. Abosabie S. A. S. Barr A. M. Mendez L. M. (2025). RalB uncoupling from exocyst is required for endothelial Weibel-Palade body exocytosis. Mol. Biol. Cell 36, mbcE24110493. 10.1091/mbc.e24-11-0493 PMC12086567 40172988

[B63] Zeng J. Shu Z. Liang Q. Zhang J. Wu W. Wang X. (2022). Structural basis of von Willebrand factor multimerization and tubular storage. Blood 139 (22), 3314–3324. 10.1182/blood.2021014729 35148377 PMC11022981

[B64] Zenner H. L. Collinson L. M. Michaux G. Cutler D. F. (2007). High-pressure freezing provides insights into Weibel-Palade body biogenesis. J. Cell Sci. 120 (Pt 12), 2117–2125. 10.1242/jcs.007781 17535847

[B65] Zhou Y. F. Eng E. T. Nishida N. Lu C. Walz T. Springer T. A. (2011). A pH-regulated dimeric bouquet in the structure of von Willebrand factor. EMBO J. 30 (19), 4098–4111. 10.1038/emboj.2011.297 21857647 PMC3209782

[B66] Zografou S. Basagiannis D. Papafotika A. Shirakawa R. Horiuchi H. Auerbach D. (2012). A complete Rab screening reveals novel insights in Weibel-Palade body exocytosis. J. Cell Sci. 125 (Pt 20), 4780–4790. 10.1242/jcs.104174 22899725

